# Combined Bennett Fracture Dislocation and Trapezium Fracture: A Rare Case Report

**DOI:** 10.7759/cureus.6211

**Published:** 2019-11-20

**Authors:** Raghavendra Kosagi Sharaph, Samundeeswari Saseendar, Saseendar Shanmugasundaram

**Affiliations:** 1 Orthopaedics, Subbaiah Hospital, Bangalore, IND; 2 Orthopaedics, Manakula Vinayagar Medical College and Hospital, Puducherry, IND; 3 Orthopaedics, Apollo Hospital, Muscat, OMN

**Keywords:** bennett fracture, trapezium fracture, wrist, fracture

## Abstract

The combination of Bennett fracture dislocation and trapezium fracture is rare and presents a diagnostic and therapeutic challenge. We report a case of a young adult male who presented with such an injury after a motor vehicle accident. He had a Bennett fracture with a vertical trapezium fracture. He was treated with K-wire fixation without a capsulotomy. The fracture healed in six weeks and at eight weeks, he regained full movement. The mechanism of this rare injury pattern is discussed. A simple modification of a commonly used treatment method resulted in faster fracture healing and recovery in the present case. The case is presented for its rarity and the modified treatment method. Higher suspicion is stressed to avoid missing the diagnosis.

## Introduction

A Bennett fracture is a well-defined injury and consists of avulsion of the attachment of the thick and strong volar oblique ligament on the base of the first metacarpal. The loss of bony stability due to the fracture, added on by the pull of the muscles attached to the first metacarpal, causes dislocation of the first metacarpal. The most common mechanism is a fall on the hand with the thumb in abduction or extension. The association of a trapezium fracture with a Bennett fracture is extremely rare and represents larger forces of injury and higher instability. We present the case of a young adult with a combined Bennett fracture dislocation and a trapezium fracture. Fixation was done without a capsulotomy to expose the fracture.

## Case presentation

A 27-year-old right-hand dominant male, a software engineer by profession, presented to the emergency department with a history of a motor vehicle accident two days prior. He complained of pain and swelling in the left thumb with a painful limitation of movements.

**Figure 1:**
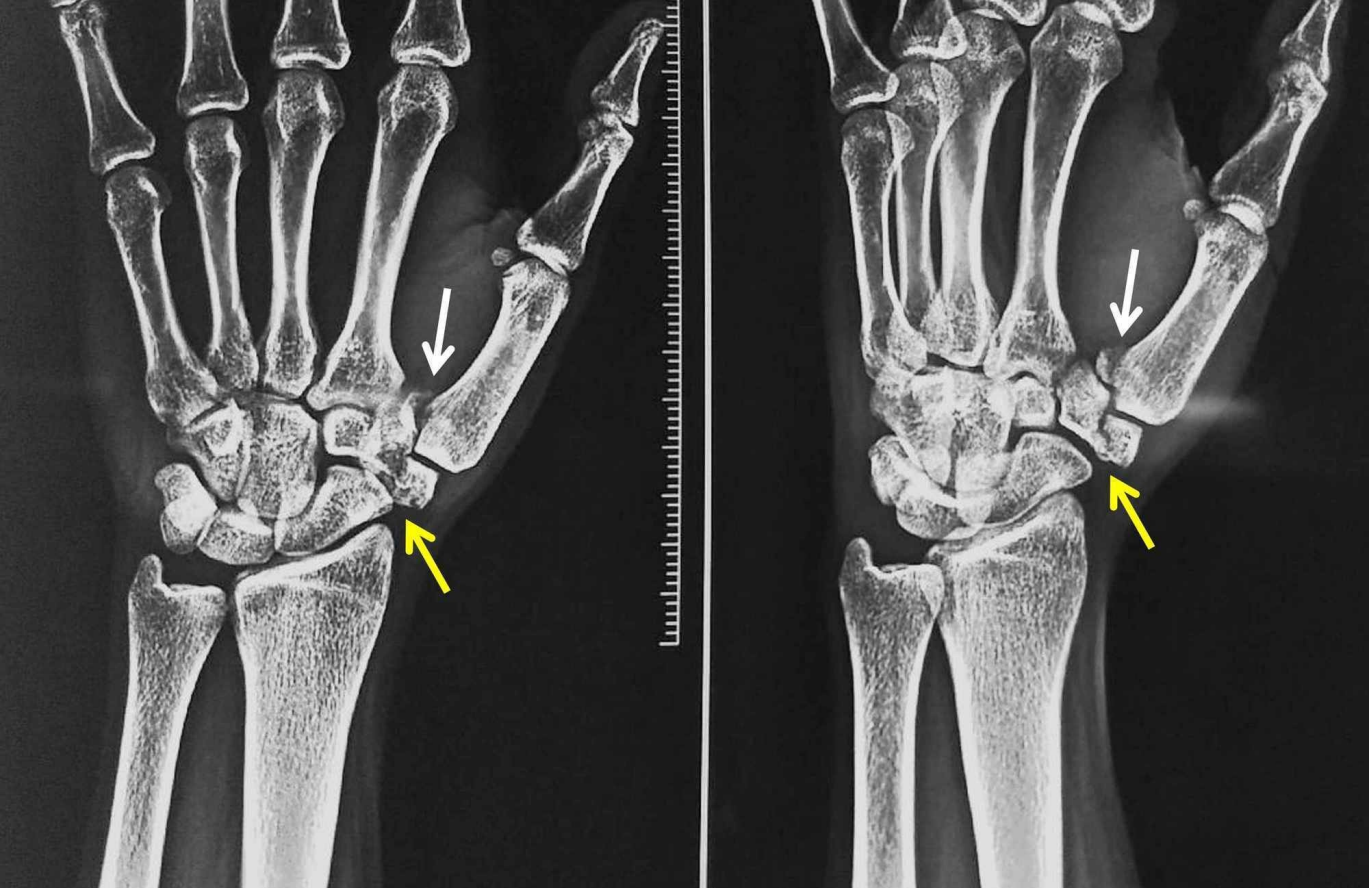
Anteroposterior and oblique radiographs of the wrist and hand White arrows - Bennett fracture; yellow arrows - trapezium fracture

Examination revealed swelling and deformity in the first carpometacarpal joint region. Active movements of the thumb were restricted due to pain. There were no external injuries. Neurovascular status was normal. Standard anteroposterior and oblique radiographs of the hand revealed a Bennett fracture along with a displaced Type IV Walker et al. fracture of the trapezium [1] with a marked intra-articular step-off (Figure 1).

**Figure 2:**
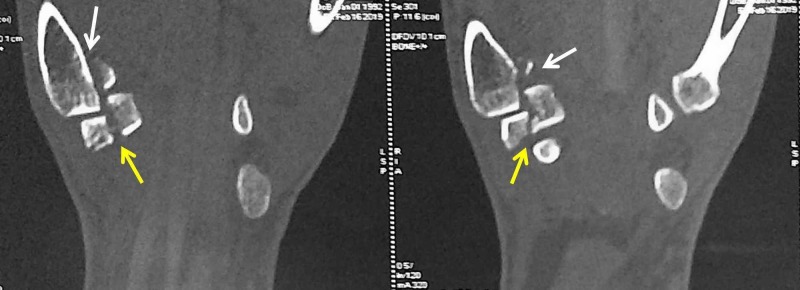
Computed tomography (coronal sections) showing Bennett fracture and trapezium fracture White arrows - Bennett fracture; yellow arrows - trapezium fracture

**Figure 3:**
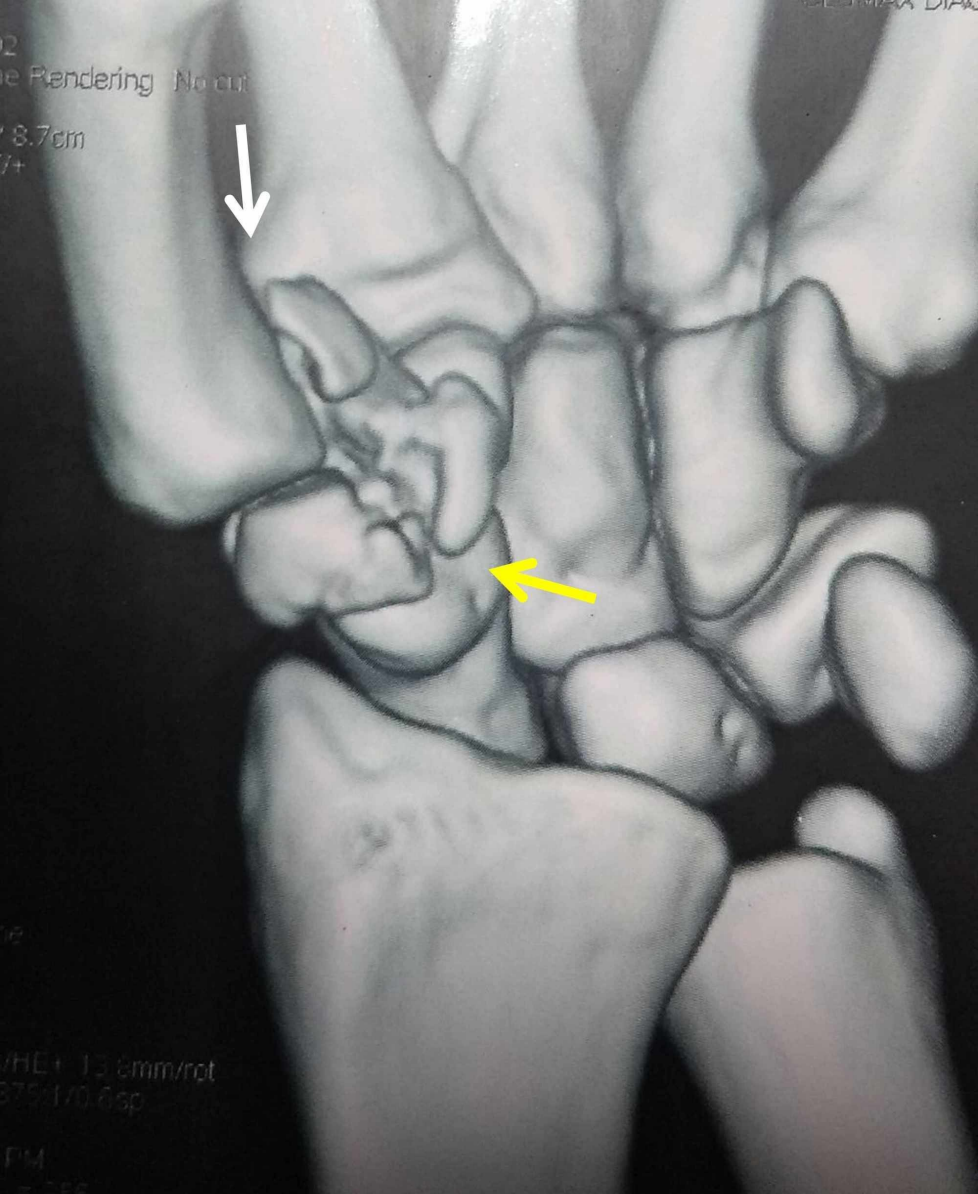
Three-dimensional computed tomography of the wrist White arrow - Bennett fracture; yellow arrow - trapezium fracture

Computed tomography confirmed the diagnosis and ruled out other concomitant carpal injuries (Figures 2-3).

**Figure 4:**
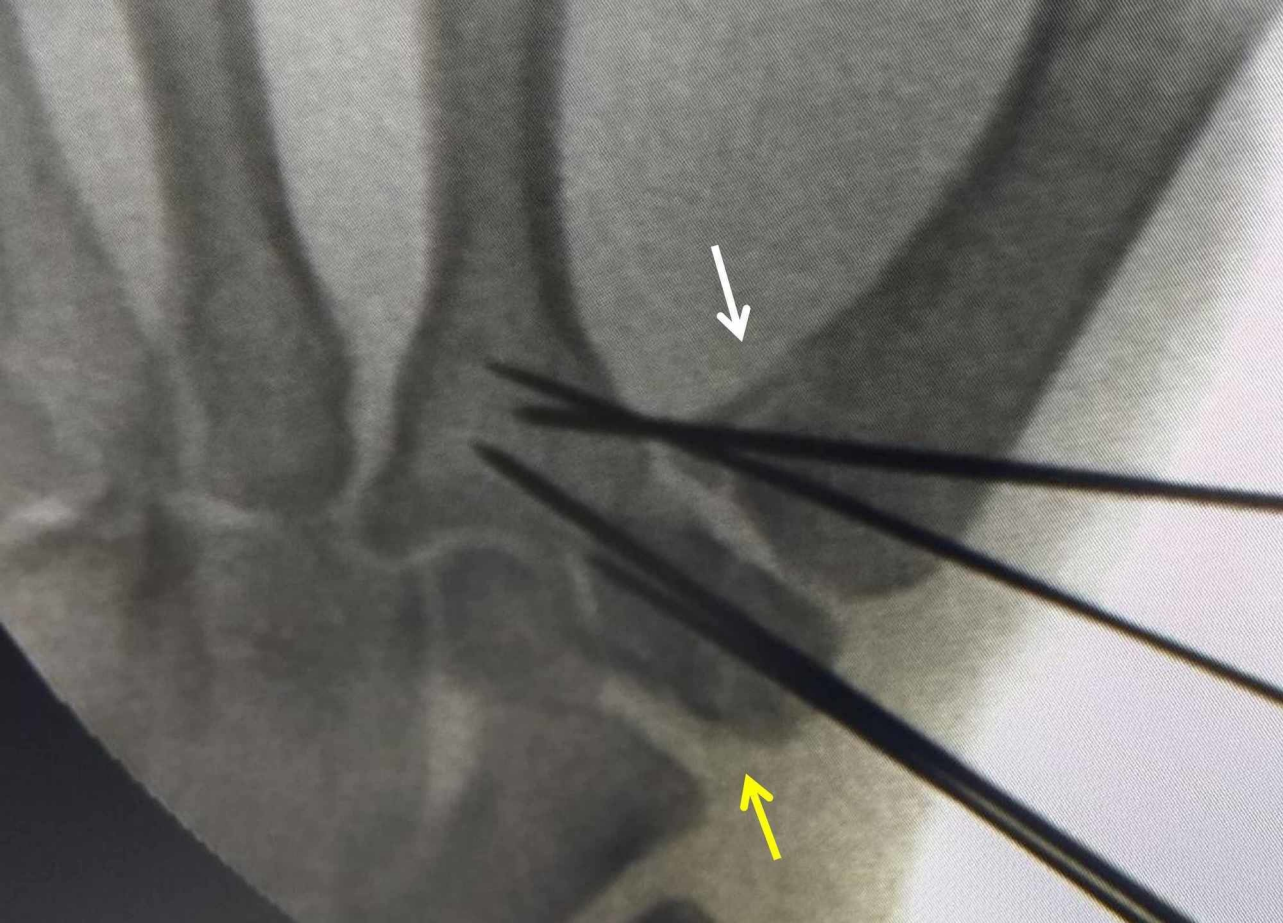
Intraoperative fluoroscopy (anteroposterior view) showing anatomical reduction of fractures White arrow - Bennett fracture; yellow arrow - trapezium fracture

**Figure 5:**
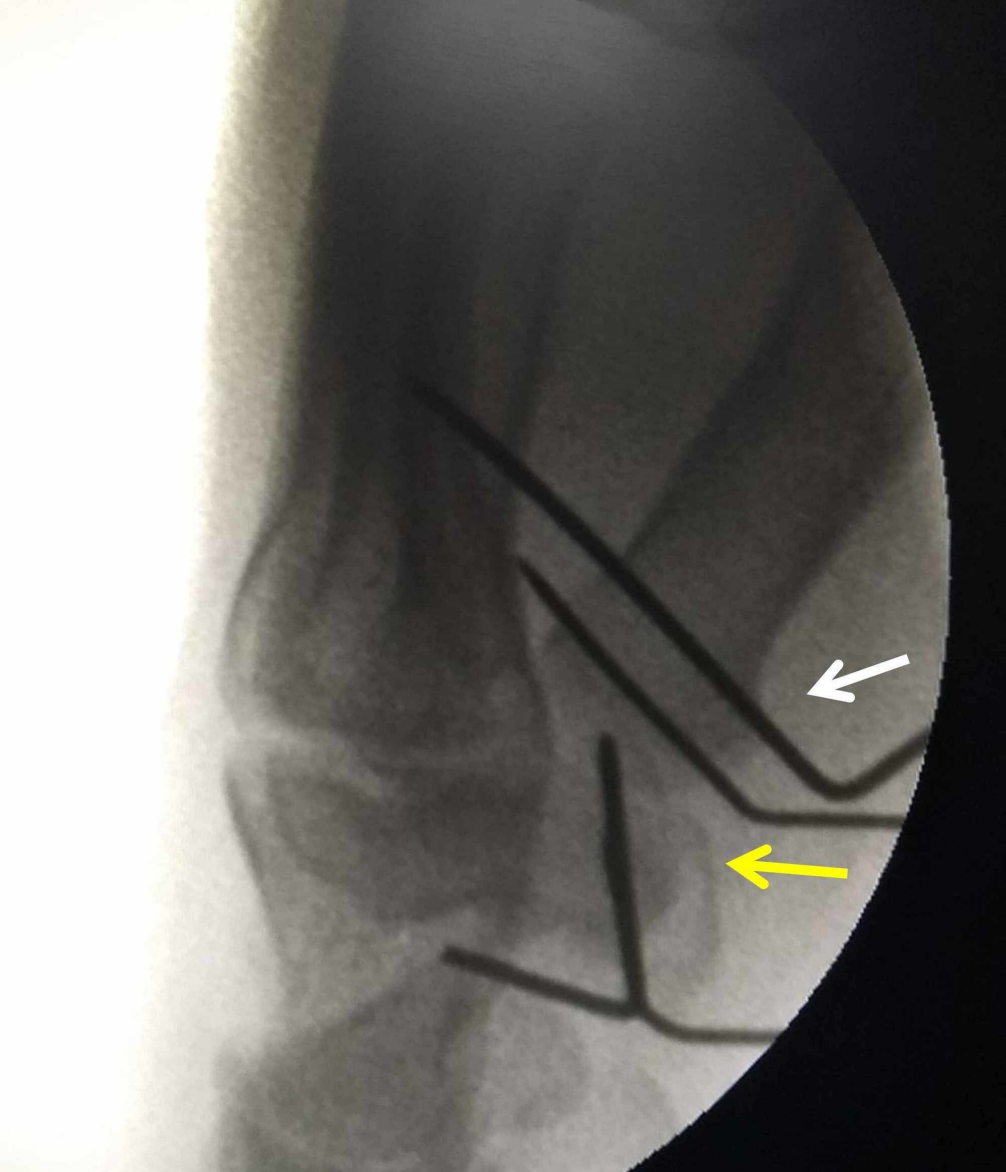
Intraoperative fluoroscopy (lateral view) showing anatomical reduction of fractures White arrow - Bennett fracture; yellow arrow - trapezium fracture

The patient was taken up for open reduction and internal fixation after two days, once the swelling reduced. Under regional anesthesia, a 3 cm linear skin incision was made centered over the first carpometacarpal joint, similar to a radio-palmar incision, along the transition zone between palmar and dorsal skin. The radial artery and sensory branches of the radial nerve were retracted and protected. The joint capsule was not opened. The trapezium fracture was reduced with mild traction and manipulation under image guidance and fixed with two 1.2 mm K-wires. The Bennett fracture was then reduced with longitudinal traction, pronation of the thumb and direct pressure over the base of the first metacarpal bone, and fixed with two 1.2 mm K-wires, crossing over to the base of the second metacarpal bone (Figures 4-5).

**Figure 6:**
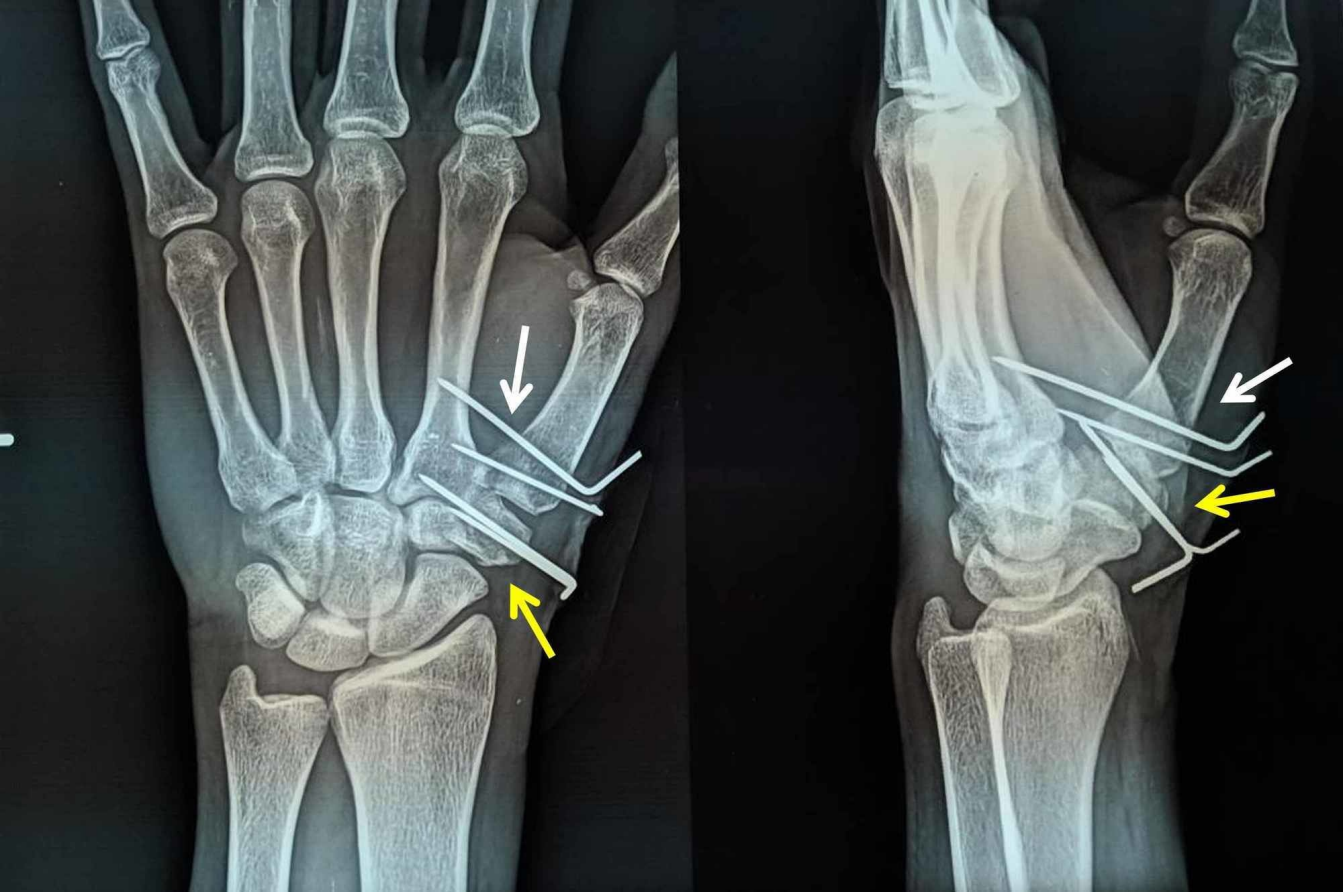
Anteroposterior and lateral radiographs of the hand at six weeks following surgery showing good fracture healing White arrows - Bennett fracture; yellow arrows - trapezium fracture

A thumb spica splint was applied postoperatively. The patient was followed up at two-week intervals. Anteroposterior and lateral wrist radiographs at six weeks showed good fracture reduction and healing (Figure 6).

The K-wires were removed at six weeks and the thumb was mobilized. The patient regained full movement of the thumb at eight weeks postoperatively. The patient was lost to follow-up subsequently.

## Discussion

Trapezium fracture accounts for only 3%-5% of all carpal bone fractures. A Bennett fracture has been associated with just 15% of all trapezium fractures [2-3].

Two different mechanisms have been proposed for the causation of a trapezium fracture along with a Bennett fracture [4-5]. In the first mechanism, a direct axial loading on a flexed thumb results in the dorsoradial subluxation of the first metacarpal, causing avulsion of the anterior oblique ligament from the volar ulnar aspect of the base of the first metacarpal. Further axial load on the metacarpal results in the impaction of the trapezium between the metacarpal and the radial styloid, resulting in a vertical split or a comminuted fracture of the trapezium.

In the second mechanism, there is an abduction force through the first web space as in sudden deceleration when driving a motorcycle or a fall when holding an object in the hand. Varying forces and varying impact angles result in a combination of a Bennett fracture-dislocation and trapezium fracture. The patient in the present case appears to have met with such an abduction force through the first web space, resulting in both a Bennett fracture dislocation and a vertical split fracture of the trapezium.

Kose et al. reviewed all reports of a combined Bennett fracture dislocation and a trapezium fracture and found that the trapezium fractures were always vertical (Walker et al. types IIa and IV) [1,4]. This is in accordance with the present case and in keeping with the mechanism of the injury.

The injury is extremely unstable and can lead to malunion, arthritis, and prolonged morbidity when left untreated [2,6]. Treatment is aimed at the restoration of the articular congruity and stable reduction of the fracture dislocation.

The trapezium fracture is fixed first in order to provide a stable base for the reduction and fixation of the carpometacarpal fracture dislocation. Open reduction of the fracture with capsulotomy of the carpometacarpal joint has often been used. However, postoperative stiffness has been reported with this method of reduction and fixation [2]. In the present case, reduction was achieved without capsulotomy under image guidance. This could have contributed to faster fracture healing and earlier achievement of full range of motion. Both K-wires and headless screws have been used in previous reports [2,5]. In the present case, K-wires were preferred to headless screws in order to avoid capsulotomy. Though K-wires can be associated with pin-tract infection or migration, early healing in these cancellous bones allows early removal and hence minimizes these risks.

The trapezium fracture can easily be missed due to a difficult clinical examination from the adjacent Bennett fracture and due to poor quality radiographs. Special views, such as the Bett view, which is a 15-degree cephalocaudal lateral view with the hand pronated 20-30 degrees, the wrist ulnar deviated and the thumb abducted, can help project all the four articulations of the trapezium without overlap from the surrounding bones [7].

## Conclusions

The combination of a Bennett fracture dislocation with a trapezium fracture is often missed and requires accurate reduction and stable fixation. A high index of suspicion is needed to avoid a missed diagnosis. Fixation without capsulotomy, whenever possible, could contribute to better stability and early recovery.
